# Potassium Dichromate Induced Cytotoxicity, Genotoxicity and Oxidative Stress in Human Liver Carcinoma (HepG_2_) Cells

**DOI:** 10.3390/ijerph6020643

**Published:** 2009-02-12

**Authors:** Anita K. Patlolla, Constance Barnes, Diahanna Hackett, Paul B. Tchounwou

**Affiliations:** Molecular Toxicology Research Laboratory, NIH-Center for Environmental Health, CSET, Jackson State University, Jackson, MS, USA; E-mails: constancepb@yahoo.com (C.B.); diahanna.m.hackett@jsums.edu (D.H.); paul.b.tchounwou@jsums.edu (P.B.T.)

**Keywords:** HepG2 cells, cytotoxicity, DNA damage, lipid peroxidation, malondialdehyde, potassium dichromate

## Abstract

Chromium is a widespread industrial waste. The soluble hexavalent chromium Cr (VI) is an environmental contaminant widely recognized to act as a carcinogen, mutagen and teratogen towards humans and animals. The fate of chromium in the environment is dependent on its oxidation state. Hexavalent chromium primarily enters the cells and undergoes metabolic reduction to trivalent chromium, resulting in the formation of reactive oxygen species together with oxidative tissue damage and a cascade of cellular events. However, the results from *in vitro* studies are often conflicting. The aim of this study was to develop a model to establish relationships between cytotoxicity, genotoxicity and oxidative stress, in human liver carcinoma [HepG2] cells exposed to potassium dichromate. HepG2 cells were cultured following standard protocols and exposed to various concentrations [0–50 μM] of potassium dichromate [K_2_Cr_2_O_7_]. Following exposure to the toxic metal, the MTT assay was performed to assess the cytotoxicity, the thiobarbituric acid test to evaluate the degree of lipid peroxidation as an indicator of oxidative stress and the alkaline comet assay was used to assess DNA damage to study genotoxicity. The results of the study indicated that potassium dichromate was cytotoxic to HepG2 cells. The LD_50_ values of 8.83 ± 0.89 μg/ml, 6.76 ± 0.99 μg/ml, respectively, for cell mortality at 24 and 48 hrs were observed, indicating a dose- and time-dependent response with regard to the cytotoxic effects of potassium dichromate. A statistically significant increase in the concentration of malondialdehyde [MDA], an indicator of lipid peroxidation, was recorded in exposed cells [15.9 – 69.9 μM] compared to control [13 μM]. Similarly, a strong dose-response relationship (p<0.05) was also obtained with respect to potassium dichromate induced DNA damage (comet assay) in HepG2 cells exposed [3.16 ± 0.70 – 24.84 ± 1.86 microns – mean comet tail length]; [12.4 ± 1.45% – 76 ± 1.49% – % tail DNA] to potassium dichromate than control [3.07 ± 0.26 microns – mean comet tail length]; [2.69 + 0.19% – % Tail DNA], respectively. The results demonstrated that potassium dichromate was highly cytotoxic to HepG2 cells, and its cytotoxicity seems to be mediated by oxidative stress and DNA damage.

## 1. Introduction

Chromium (Cr) is a naturally occurring heavy metal commonly found in the environment in two valence states: trivalent Cr (III) and hexavalent Cr (VI). It is widely used in numerous industrial processes and as a result, is a contaminant of many environmental systems [1]. Commercial chromium compounds are used in industrial welding, metal finishes, leather tanning and wood preservation and it is a non-negligible pollutant in the world [2,3]. Studies in animal models also found many harmful effects of Cr (VI) on mammals. Subcutaneous administration of Cr (VI) to rats caused severe progressive proteinuria, urea nitrogen and creatinine, as well as elevation in serum alanine aminotransferase activity and hepatic lipid peroxide formation [4]. Similar studies reported by Gumbleton and Nicholls [5] found that Cr (VI) induced renal damage in rats when administered by single sc injections. Bagchi *et al*. demonstrated that in rats Cr (VI) received orally in water induced hepatic mitochondrial and microsomal lipid peroxidation, as well as enhanced excretion of urinary lipid metabolites including malondialdehyde [6,7]. Moreover, some adverse health effects induced by Cr (VI) have been reported in humans. Reports of epidemiological investigations have shown that respiratory cancers have been found in workers occupationally exposed to Cr (VI) compounds [8,9]. DNA strand breaks in peripheral lymphocytes and lipid peroxidation products in urine observed in chromium exposed workers in many researches also showed evidence of the Cr (VI)-induced toxicity to humans [10,11].

The carcinogenicity of specific chromium compounds is influenced by both the valence and the solubility of the chromium species. Chromium (VI) compounds have been reported to be more toxic and carcinogenic than chromium (III) ones [12,13] because the former can pass through cell membranes more easily than the latter [14]. Once inside the cell, Cr (VI) is reduced to its lower oxidation states [Cr (V)] and [Cr (IV)] and then Cr (III) by low molecular weight molecules, enzymatic and non-enzymatic reductants [15]. These reactive chromium intermediates are capable of generating a whole spectrum of reactive oxygen species (ROS), which is an important characteristic of Cr (VI) metabolism [16]. Excessive quantities of ROS generated by these reactions can cause injury to cellular proteins, lipids and DNA leading to a state known as oxidative stress [17]. Therefore, one of the most important damages caused by extraneous Cr (VI) is massive production of ROS during the reduction of Cr (VI) in the cell.

Lipid peroxidation (LPO), the oxidative catabolism of polyunsaturated fatty acids, is widely accepted as a general mechanism for cellular injury and death [18,19]. LPO and free radical generation are complex and deleterious processes that are closely related to toxicity [20]. LPO has been implicated in diverse pathological conditions, including atherosclerosis [21], aging [22], rheumatoid arthritis [23], and cancer [24]. It is also involved in the toxicity of pesticides [25], solvents [26] and metals [27]. The extension of the oxidative catabolism of lipid membranes can be evaluated by several endpoints, but the most widely used method is the quantification of malondialdehyde (MDA), one of the stable aldehydic products of lipoperoxidation, present in biological samples [28,29].

Although chromium and chromium-containing compounds has been the subject of important toxicology research, there exists a lack of appropriate *in vitro* model to understand the mechanism for cytotoxic effects, oxidative stress, and DNA damage. There is also scarcity of scientific data with respect to their toxicity in *in vitro* systems. Therefore, the present work was undertaken to study the role of cell proliferation, oxidative stress and DNA damage in HepG_2_ cells exposed to hexavalent chromium.

## 2. Materials and Methods

### 2.1. Chemicals

Potassium dichromate, sodium chloride, sucrose, Triton^®^ X-100, hydrochloric acid, histopaque-1077, NaOH, ethanol, trypan-blue and EDTA were obtained from Sigma-Aldrich (St. Louis, MO, USA). They were of analytical grade or highest grade available. The Lipid Peroxidation (LPO) assay kit was purchased from Calbiochem (San Diego, CA, USA). Dulbecco’s Modified Eagle’s Minimal Essential Medium (DMEM), Phosphate buffer (pH 7.4), trypsin-EDTA, penicillin, streptomycin, fetal bovine serum (FBS) were obtained from GIBCO (New York, NY, USA). Comet assay kit was purchased from Trevigen, Inc., (Gaithersburg, MD). Human liver carcinoma, HepG_2_ cells were purchased from ATCC (Manassas, VA). Sterile Tissue culture flask and sterile glass pippets were purchased from Fischer-Scientific.

### 2.2. Cell Culture and Cytotoxicity

Parental HepG_2_ cells stored in liquid nitrogen were thawed by gentle agitation of their containers (vials) for 2 min in a water bath at 37 °C. After thawing, the content of each vial was transferred to a 75 cm^2^ surface area, tissue culture flask, diluted with DMEM supplemented with 10% fetal bovine serum (FBS) and 1% streptomycin and penicillin. Then the cells are incubated for 24 h at 37 °C in a 5% CO_2_ incubator to allow the cells to grow, and form a monolayer in the flask. Cells grown to 80–95% confluency were washed with phosphate buffer saline (PBS), trypsinized with 3 mL of 0.25% (v) trypsin-0.03%/v) EDTA, diluted, counted and seeded (5 × 10^5^ cells/well) in two sets of 96-well microtiter tissue culture plates.

Seeded plates were incubated for 24 h at 37 °C in a 5% CO_2_ incubator. The old medium was replaced by 180 μL of fresh medium. Twenty micro liters of serial dilutions of potassium dichromate (0, 3.125, 6.25, 12.5, 25 and 50 μM) were added column wise to the 96-well microtiter tissue culture plates and incubated for 24 and 48 h exposure time. Cell viability assay was performed using the MTT {3-(4,5-dimethylthiazol-2-yl)-2,5-diphenyl-tetrazolium bromide} method. The absorbance was read at a wavelength of 550 nm using microtiter plate reader (Bio-Tek Instruments Inc.).

### 2.3. ROS Detection

ROS production was quantified by the DCFH-DA method [30] based on the ROS-dependent oxidation of DCFH-DA to DCF. An aliquot of cell suspension from each of the four treated and the control was centrifuged at 1000x g for 10 min (4 °C). The supernatants were re-centrifuged at 20,000x g for 20 min at 4 °C, and then the pellet was re-suspended. The DCFH-DA solution with the final concentration of 50 μM and re-suspension were incubated for 30 min at 37 °C. Fluorescence of the samples was monitored at an excitation wavelength of 485 nm and an emission wavelength of 538 nm.

### 2.4. Comet Assay

DNA damage was determined in HepG_2_ cells exposed to four doses of potassium dichromate and control for 48 h using comet assay kit from Trevigen (Gaithersburg, MD). Following isolation the cells were mixed with 0.4% Trypan blue solution, after 15–20 min cells were counted and checked for viability. The remaining cells were immediately used for single-cell gel electrophoresis. The assay was performed according to Singh *et al.* [31] with slight modifications. All the steps were conducted under yellow lamp in the dark to prevent additional DNA damage. Stained Slides are viewed under automated robotic epiflorescent microscope. A total of 150 individual cells were screened/sample [duplicate, each with 75 cells. Slides were read using DNA damage analysis software [Loats Associates Inc., Westminster, MD]. Each experiment was performed in triplicate. Cells with damaged DNA displayed high migration of DNA fragments from the nucleus, forming a tail in comet form. Most important parameters besides mean comet tail length that were used for analyzing genotoxicity in the comet assay were:

DNA head (DNAH), sum of intensities of all points of the head.
DNA tail (DNAT), sum of intensities of all points of the tail.
Percent tail DNA (%DNAT) = 100DNAT/(DNAH+DNAT)

### 2.5. Malondialdehyde (MDA) Determination

Malondialdehyde concentration was measured in HepG_2_ exposed to four doses of potassium dichromate and a control for 48 h using lipid peroxidation assay kit from Calbiochem (San Diego, CA, USA). Briefly, 0.65 mL of 10.3 mM N-methyl-2-phenylindole in acetonitrile was added to 0.2 mL of sample. After vortexing for 3–4 sec and adding 0.15 mL of 37% HCl, samples were mixed well and closed with a tight stopper and incubated at 45° C for 60 mins. The samples were then cooled on ice, centrifuged and the absorbance was measured spectrophotometrically at 586 nm. A calibration curve of an accurately prepared standard MDA solution (from 2–20 nmol/mL) was also run for quantification. Measurements of each group were performed in triplicate. The standard deviations were less than ± 10%.

## 3. Data Analysis and Statistics

Data were compared by ANOVA. Statistical analysis was performed using SAS for Windows 2003 package program. Using the Dunnett test, multiple comparisons were performed. All values were reported as means ± SD for all the experiments. The significance level was set at *p*<0.05.

## 4. Results

### 4.1. Cytotoxicity Assay

**Figure 1.**
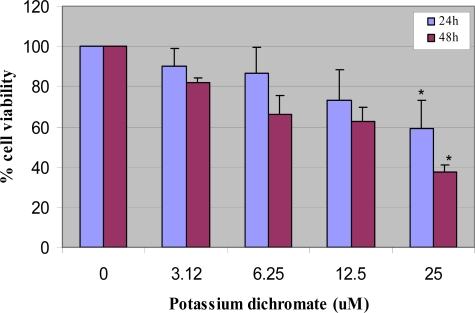
Cytotoxicity after 24 and 48 h exposure to potassium dichromate in HepG2 cells using MTT assay. Each experiment was done in triplicate. Data were represented as means ± SDs. Statistical significance was indicated as (*) for (p< 0.05).

The MTT result of the cytotoxic effect of potassium dichromate on human liver carcinoma cells following 24 and 48 h of exposure is shown in Figure 1. This figure indicates a strong dose- and time-dependent response relationship with respect to chromium toxicity. After 48 h of exposure, the percentages of cell viability were 100 ± 0.0, 82.0 ± 12.0, 55.0 ± 5.8, 40.0 ± 12.0, and 35.0 ± 3.7% for the control, 3.12, 6.25, 12.5 and 25 μM of potassium dichromate respectively. The dose of potassium dichromate required to produce 50% reduction in the viability of HepG_2_ cells (LD_50s_) were compound to be 8.83 ± 3.5 and 6.76 ± 0.7 μg/mL upon 24 and 48 h of exposure, respectively; indicating a dose- and time-dependent response with regard to the cytotoxic effect of potassium dichromate.

### 4.2. ROS Detection

**Figure 2.**
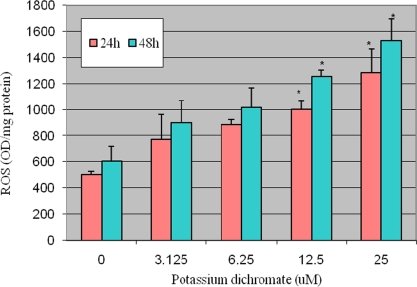
Potassium dichromate induced ROS in HepG2 cells. Each experiment was done in triplicate. Data were represented as means ± SDs. Statistical significance was indicated as (*) for (p< 0.05).

ROS were determined in control and exposed groups after administration of potassium dichromate to HepG_2_ for 48 h. The exposure of Cr (VI) to HepG_2_ cells significantly enhanced the ROS level at four tested doses as compared to the control group and increases were found to show dose-dependent relationship. Figure2. Represent the result of ROS detection.

### 4.3. DNA Damage [Comet assay]

**Figure 3.**
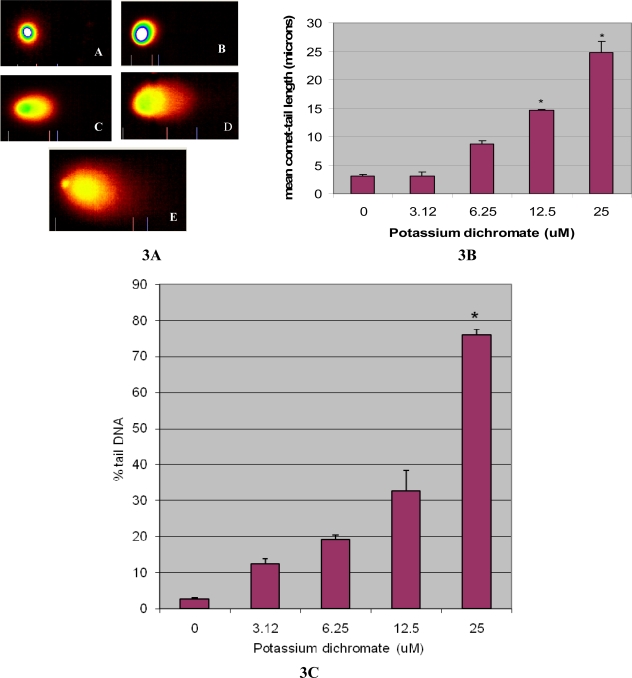
(A) Single Cell Gel Electrophoresis assessment of potassium dichromate toxicity in human liver carcinoma cells (HepG2): A) Representative Comet images of control (A), and 3.12 μM (B); 6.25 μM (C); 12.5 μM (D) and 25 μM (E); 3(B): Effect of various doses of potassium dichromate and control on DNA migration in HepG_2_ cells at 48 h exposure; 3(C): Effect of various doses of potassium dichromate and control on the percent tail DNA. Each experiment was done in triplicate. Data were represented as means ±SDs. Statistical significance was indicated as (*) for (p< 0.05).

Comet tail length is an important parameter in evaluating the DNA damage. All the doses of potassium dichromate induced statistically significant increase in mean comet tail length [3.16 ± 0.7 – 24.8 ± 1.86 microns] indicating DNA damage when compared with controls [3.07±0.26 microns]. Maximum increase in mean comet tail length was observed at 25 μM at 48h post-treatment [24.8 microns]. Besides the mean comet tail length, percent tail DNA was also measured in the exposed and control cells. The following percent tail DNA 12.4 ± 1.45%, 19.2 ± 1.16%, 32.6 ± 5.79 and 76 ± 1.49% respectively was observed in the HepG2 cells exposed to various concentration of potassium dichromate compared to control 2.69 ± 0.19%. The mean comet tail length showed a clear dose-dependent increase from 3.12 to 25 μM. The results of DNA damage cells are illustrated in Figure 3(A) (B) and (C) respectively. Representative comet assay images of control (A) and potassium dichromate treated HepG_2_ cells at 3.12 μM (B); 6.25 μM (C); 12.5 μM (D) and 25 μM (E) are presented in Figure3A. Genotoxicity as characteristized by the length of the comet tail is illustrated in Figure 3B.

### 4.4. Lipid Peroxidation

**Figure 4.**
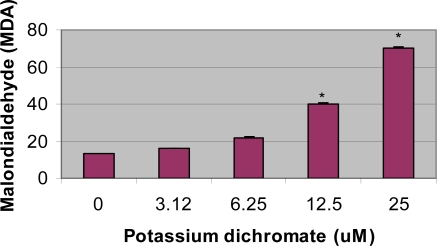
Induction of lipid peroxidation in HepG2 cells by various doses of potassium dichromate and control. Each experiment was done in triplicate. Data were represented as mean + SD. Statistical significance was indicted as (*) for (p <0.05).

One of the methods for evaluating lipid peroxidation is measurement of malondialdehyde concentration in the cells. Potassium dichromate exposure significantly *(p<0.05*) increased the concentration of MDA in HepG_2_ cells when compared with the control. Results are illustrated in Figure 4. The increase in MDA concentration in HepG_2_ was found to be dose-dependent, indicating a gradual increase with increasing dose of potassium dichromate. Statistically significant increase in the concentration of malondialdehyde [MDA], an indicator of lipid peroxidation, was recorded in exposed cells [15.9 – 69.9 μM] compared to control [13 μM].

## 5. Discussion

In the present study concentration- and time-dependent effects of potassium dichromate were assessed on the reductions of tetrazolium dye MTT in cultured HepG_2_ cells. This is a marker of succinate dehydrogenase activity, an index of the mitochondrial electron transport system, which is used to assess cell viability. Only viable cells with intact mitochondria can reduce tetrazolium dye MTT and the amount of MTT reduced is directly proportional to the number of viable cells present. Data obtained from the present study, clearly indicate that potassium dichromate is highly cytotoxic to human liver carcinoma cells. The LD_50_ were computated to be 8.83 ± 3.5 and 6.76 ± 0.7 μg/mL after 24 and 48 h of exposure, respectively; indicating a dose- and time-dependent response with regards to the cytotoxic effect of potassium dichromate. These results support those of a previous investigation reporting a marked reduction in the viability of K562 and J774 cells exposed to chromium (VI) [32].

In this study we observed that there was a significant increase in the level of ROS in HepG_2_ cells exposed to potassium dichromate compared to control. Reactive oxygen species (ROS) have been implicated in the toxicity of chromium (VI) by several authors [7,15,33]. Their formation with subsequent cellular damage is considered as the common molecular mechanism of Cr (VI)-induced toxicity. According to this hypothesis, chromium (VI) itself is not a cytotoxic agent but rather an oxygen free radical generator through cellular reduction to chromium (VI) [34]. Chromium reduction intermediates are believed to react with hydrogen peroxide to form the hydroxyl radical [35], which may finally attack proteins, DNA, and membranes lipids thereby disrupting cellular functions and integrity [7].

In the present study, measuring the concentration of MDA in HepG_2_ of potassium dichromate-exposed and control group assessed lipid peroxidation. There was a significant increase in the concentration of MDA after 48 h of potassium dichromate administration. These results are in accordance with those obtained by Bagchi *et al.* [32], who detected oxidative lipid metabolites in K562 and J774 cells exposed to Cr (VI). The increase observed in lipid peroxidation may be due to the formation of hydroxyl radical (HO) through a Fenton/Haber-Weiss reaction, catalysed by chromium. This radical is capable of abstracting a hydrogen atom from a methylene group of polyunsaturated fatty acids enhancing lipid peroxidation.

The frequency of single-strand breaks showed a clear dose-related increase in HepG_2_ cells up to 25 μM in our investigation. Maximum DNA damage was observed at 48 h post-treatment when compared with the controls. Similar results were reported with other heavy metals in rodents and in other cell-lines using comet assay [3,36]. Cr (VI) itself is not reactive to DNA, however, the chromium metabolites radicals produced during reduction can subsequently attack macromolecules and lead to multiform DNA damages e.g. strand breakage, DNA-protein cross-links, DNA-DNA cross-links, Cr-DNA adducts and base modifications in cells. Especially DNA strand breaks are mainly ascribed to the ROS [3,37,38]. Single cell gel electrophoresis (comet assay) is a highly sensitive technique to evaluate single strand breaks and alkali labile sites in DNA of individual cells.

In summary, the current study demonstrates that administration of Cr (VI) to human liver carcinoma cells for 48 h could induce cytotoxicity, DNA damage and oxidative stress. ROS may play an essential role in DNA damage and oxidative stress induced by Cr (VI) *in vitro*. Our results show that the use of thiobarbituric acid reactive substances (TBARS) as a marker of oxidative stress should be complemented with antioxidant parameters namely SOD and CAT. The results support an involvement of the oxidative damage pathway in the mechanism of toxicity of chromium. Further studies of the behavior of the antioxidant enzymes can aid in the understanding of chromium-induced toxicity.
